# The Effect of Mental Fatigue on Neuromuscular Function is Similar in Young and Older Women

**DOI:** 10.3390/brainsci10040191

**Published:** 2020-03-25

**Authors:** Amanda J. Morris, Anita D. Christie

**Affiliations:** 1 Department of Human Physiology, University of Oregon, Eugene, OR 97401, USA; amorris8@uoregon.edu; 2 School of Kinesiology, Western University, London, ON N6A 3K7, Canada

**Keywords:** mental fatigue, neuromuscular function, aging

## Abstract

The purpose of this study was to examine the effect of a mentally fatiguing task on neuromuscular function in young and older women. Neuromuscular measures were obtained prior to and following 20 min of a mentally fatiguing task. Maximal force output significantly decreased after the mental fatigue task (*p* = 0.02) and this was not different between age groups (*p* = 0.32). Increases in cortical silent period duration approached significance in both young and older groups (*p* = 0.06), suggesting that mental fatigue may cause increased cortical inhibition. Measures of peripheral neuromuscular function (contractile properties of the muscle, M-wave) did not change (*p* ≥ 0.09), suggesting that changes in force production with mental fatigue are more likely due to supraspinal than peripheral mechanisms. These findings provide further evidence of an interaction between mental fatigue and physical function.

## 1. Introduction

Mental fatigue is a psychophysiological state that occurs after or during prolonged periods of cognitive activity [1]; it is a component of subjective fatigue and is characterized by self-reported feelings of tiredness, lack of motivation, and decreased cognitive performance [2,3]. Several studies have examined the effect of mental fatigue on exercise performance and the perception of fatigue. Decreases in endurance time and loss of peak power during cycling in the presence of mental fatigue have been reported [4,5], as well as decreases in running velocity, running distance, and accuracy of a soccer shot [6,7]. Furthermore, several studies using single joint exercises in the presence of mental fatigue have reported reductions in time to exhaustion and maximal force production [8,9]. All of these studies reported increases in ratings of perceived exertion with mental fatigue. Although these results suggest a negative effect of mental fatigue on physical performance and perception of exertion, and the mechanisms leading to these deficits remain unclear.

Although several studies have found no effect of mental fatigue on muscle contractile properties or maximal force production [9,10,11], conflicting reports do exist [12]. As suggested in a recent review [13] further work is necessary to truly understand the impact of mental fatigue on the neuromuscular system. Additionally, assessments of neuromuscular function in response to mental fatigue have been largely limited to peripheral measures, with few assessments of changes in central factors, such as motor cortex function, which may offer important mechanistic insights.

The effects of mental fatigue on exercise performance and neuromuscular function may vary based on sex [13] and/or aging [14]. On subjective reports, women indicate significantly more mental and physical fatigue than men [15,16,17]. Older adults, especially older women, also report higher levels of subjective fatigue than young adults [17]. In older adults, self-reported fatigue is associated with earlier onset of disability, slower gait speed, and increased hospitalization risk [14,18]. Despite these important associations, little is known about the direct impact of mental fatigue on neuromuscular function in the older population. Additionally, numerous physiological changes take place with aging, including: declines in sensory system feedback, nerve conduction velocity, motor unit numbers, muscle mass, and central processing abilities [19]. The impact of mental fatigue on neuromuscular function may differ in older adults compared to young due to the age-related changes in both neuromuscular and cognitive processes. Although mental fatigue is highly reported in older adults, to our knowledge there are no studies examining the possible age-related differences in effects of mental fatigue on neuromuscular performance. Information about the interactions between cognitive and motor functions and how these interactions change with age is important for our understanding of healthy aging. Such information will be essential in developing evidence-based interventions to improve function and prevent falls in older adults.

The purpose of this study was to examine the effect of a mentally fatiguing task on neuromuscular functions in young and older women. Neuromuscular measures were obtained prior to and following a mentally fatiguing task. It was hypothesized that the mentally fatiguing task would negatively affect neuromuscular function in both age groups, with older women showing greater changes compared to the younger group.

## 2. Materials and Methods

### 2.1. Participants 

Nine young women and 16 older women (Table 1) participated in this study. Participants were healthy and free from any chronic disease, illness, condition, or medication that could impact balance. Exclusion criteria included: a positive screen for cognitive impairment as indicated by the Mini-Cog test, history of illness associated with fatigue (e.g., chronic fatigue syndrome, multiple sclerosis), history of cognitive deficiencies (e.g., memory loss, difficulty concentrating), sleeping disorder, history of neurological impairment, history of musculoskeletal impairments, use of alcohol or central nervous system depressant pharmacological agents within 12 hours of performing tasks, or active substance abuse. All participants provided written informed consent and were asked to complete a brief medical history report, Pittsburgh Sleep Quality Index, Multidimensional Fatigue Index, and the Mini-Cog cognitive screening test. The procedures were reviewed and approved by the University of Oregon’s Institutional Review Board (IRB# 07122016.012). 

**Figure 1:**
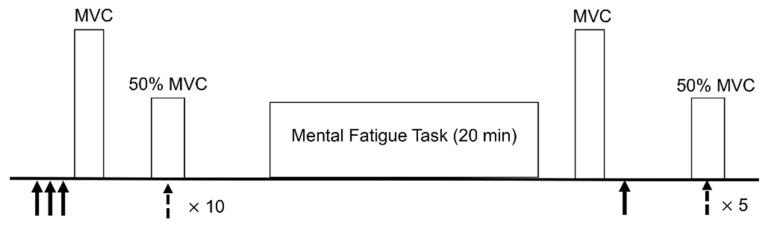
Overview of experimental timeline. Black solid arrow: electrical stimulation. Black dashed arrow: transcranial magnetic stimulation. Subjective ratings of fatigue were provided before and after the protocol.

After completing sleep and fatigue questionnaires, neuromuscular measures (electrical nerve stimulation and transcranial magnetic stimulation (TMS)) were taken. Participants then performed a mentally fatiguing task for 20 min. The neuromuscular measures were repeated immediately following the mental fatigue task. The timeline for the experimental protocol is presented in Figure 1.

### 2.2. Questionnaires

Sleep quality and fatigue state were assessed to characterize the young and older participants, as both sleep and fatigue state have been shown to be associated with mental fatigue [20]. The Pittsburgh Sleep Quality Index (PSQI) was used to measure the sleep patterns and quality of sleep in seven domains subjective sleep quality, sleep latency, sleep duration, habitual sleep efficiency, sleep disturbances, sleep medication use, and daytime dysfunction. The participants self-rated these aspects of sleep quality on a 0 to 3 scale, where 3 is negative. A total score, summed across all questions, of 5 or greater indicates a poor sleeper [21].

The Multidimensional Fatigue Inventory (MFI) is a 20 item, self-report instrument that measures fatigue in the following domains: general fatigue, physical fatigue, mental fatigue, motivation, and activity. Participants responded to each item on a scale of 1 to 7 and the total score was summed across all items. A higher total score corresponds to more acute levels of fatigue.

### 2.3. Neuromuscular Measures 

Individuals were seated in a chair with their dominant foot placed in a custom-built device designed to measure dorsiflexion force. A strap was placed over the dorsum of the foot. Maximal voluntary contraction (MVC) force was determined by participants pulling their foot against the strap as hard as they could for 5 s. They were asked to repeat this procedure an additional two times and were given at least 1 min of rest between contractions. The highest value was taken as the MVC. 

A preamplified, bipolar Ag-AgCl electrode (DE-2.1, Delsys Inc., Boston, MA, USA), with an inter-electrode distance of 1 cm, was taped to the surface of the skin, over the belly of the tibialis anterior (TA) muscle. This electrode was connected to a portable amplifier (Delsys Inc., Boston, MA, USA), which further amplified and band-pass (20–450 Hz) filtered the signal. A ground electrode was applied to the ankle. The signal was sampled at 1 kHz with a 16-bit A/D converter (NI USB-6251, National Instruments, Austin, TX, USA). 

The maximal electrical response of the muscle (M-wave) was determined by placing a stimulating electrode on the side of the leg over the peroneal nerve and activating the nerve through brief (200 μs) electrical pulses. The intensity required to elicit a maximal M-wave (recorded with the EMG electrodes) was determined by increasing the intensity of stimulation until a further increase did not result in an increase in the peak-to-peak amplitude of the M-wave. To ensure maximal stimulation throughout the protocol, the stimulator was then set to 120% of the determined maximal intensity. Electrical (M-wave) and mechanical (twitch force) responses to three stimulations at this intensity were then recorded to establish baseline measures, and an additional stimulation was provided following performance of the psychomotor vigilance task (PVT) (Figure 1). M-wave peak-to-peak amplitude was used to examine electrical response of the muscle. Force responses to stimulations were used to examine contractile properties of the muscle and included: peak twitch force, time to peak twitch force, and half-relaxation time. All data were analyzed with custom-written programs using MatLab software (MathWorks Inc., Natick, MA, USA). Peak twitch force was the maximal force generated during the twitch. Time to peak twitch force was calculated as the time from the beginning of the response to peak force. Half-relaxation time was considered as the time from peak twitch force to the time force relaxed to 50% of the peak twitch force.

Single-pulse transcranial magnetic stimulation (TMS) was used to assess the impact of mental fatigue on the excitability and inhibition of the corticospinal pathway. TMS stimuli were delivered using a 110 mm double cone magnetic stimulation coil placed on the head, over the motor cortex. This coil was used to activate the brain through brief magnetic pulses (100 μs). The response of the TA muscle was recorded with EMG. The optimal site for stimulation of the TA muscle was determined by moving the coil to find the location that presented the largest motor evoked potential (MEP) at 60% stimulator output. The resting motor threshold (RMT) of the muscle was determined by stimulating at decreasing stimulus intensity to find the threshold while the muscle is at rest. Threshold was defined as the stimulus intensity that produced a MEP of at least 50 μV in at least 5/10 trials [22]. The stimulus intensity was then set at 120% of RMT and responses were recorded while the participant was contracting at 50% MVC. Ten responses, separated by 2–3 s, were recorded before the mental fatigue task. To ensure all measurements were obtained in a short amount of time following the mental fatigue task, 5 responses were recorded after the PVT, which has been shown to produce a reliable measure of MEP amplitude and cortical silent period (CSP) duration [23]. Using custom-written MatLab programs, cortical excitability was determined by the peak-to-peak amplitude of the active MEP, averaged across trials at each time point. Cortical inhibition was determined by the cortical silent period, measured as time between the end of the MEP and resumption of voluntary EMG activity, averaged across trials at each time point. 

### 2.4. Mental Fatigue Task

To induce mental fatigue, participants were asked to perform the psychomotor vigilance task for twenty minutes. The PVT is an objective, valid measure for assessing behavioral alertness and vigilant attention [24].The PVT is based on simple reaction time to stimuli that occur randomly [24]. Lapses in reaction time (RT > 500 ms) during this task are associated with subjective measures of physical fatigue and decline in energy [25,26]. When the task is performed for 20 min or more a time-on-task effect of increase in reaction time and/or decrease in accuracy over the task is observed, indicating a decrease in vigilance and presence of mental fatigue [2,3,27]. Based on this previous work, 20 min of the PVT was used in the current study. 

Participants were asked to visually fixate on a computer screen placed at eye level in front of them. They were asked to click the left button on a mouse as soon as a red number appeared on the screen. As soon as the button was pushed, a number was displayed on the screen for 500 ms indicating reaction time and was then cleared and the next stimulus presented. Participants were instructed to keep the number as low as possible. Time between presentation of each stimulus varied randomly between 2 and 10 s. In addition to simple reaction time (RT), the program recorded: false starts, anticipation (RT < 100 ms), minor lapses (RT ≥ 500 ms), and major lapses (RT ≥ 1000 ms), which were then averaged over the first (baseline) and final (mental fatigue) 5 min. Increases in reaction time and/or number of lapses indicated the presence of mental fatigue. Before and immediately following completion of the PVT, participants are also asked to report their subjective level of fatigue on a scale of 1 to 10, with higher numbers indicating greater feelings of fatigue. This software was created by Biotechnology HPC Software Applications Institute and is run using MATLAB (MathWorks Inc., Natick, MA, USA). 

### 2.5. Statistical Analyses

All statistical analyses were performed with IBM SPSS Statistics for Windows (IBM Corp., Armonk, NY, USA). All data were normally distributed, as determined with Shapiro–Wilk tests for each outcome variable. The following quantitative outcome variables were obtained: mental fatigue: reaction time, number of lapses; neuromuscular function: MVC force, peak-to-peak amplitude of the M-wave; contractile properties of the muscle: time to peak force, peak force, half-relaxation time; cortical excitability: peak-to-peak amplitude of active motor evoked potential; cortical inhibition: cortical silent period duration. 

Participant characteristics and all baseline measures were compared with independent samples t-tests. To examine the impact of the mental fatigue protocol on each variable, 2-factor (age and time) repeated-measures analyses of variance (ANOVAs) were used. If significance was found, pairwise comparisons with a Bonferroni correction were used for post-hoc testing. Significance was set at *p* ≤ 0.05. Furthermore, effect sizes were calculated as Cohen’s d for all effects of time and age. All data are presented as mean ± SD.

## 3. Results

### 3.1. Participant Characteristics

Participant characteristics and scores for the Multidimensional Fatigue Inventory (MFI) and the Pittsburgh Sleep Quality Index (PSQI) are presented in Table 1. There was no significant difference in height (*p* = 0.44), weight (*p* = 0.50), PSQI score (*p* = 0.40) between age groups. Older women had significantly higher MFI scores (*p* = 0.03), but lower subjective rating of fatigue (*p* = 0.05) than younger women at baseline. 

**Figure 2:**
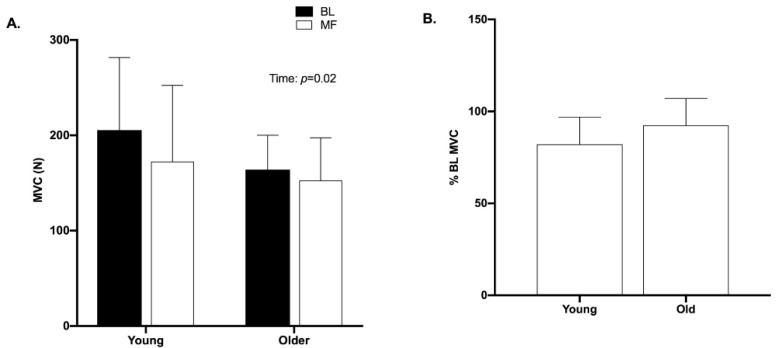
Baseline (BL) and mental fatigue (MF) maximal voluntary contraction (MVC) force values. (**A**) There was a significant decrease (*p* = 0.02) in MVC with MF. (**B**) MVC force after MF protocol. Data presented as percent of BL MVC after MF.

There was no significant difference between groups in baseline MVC force (*p* = 0.17) (Figure 2). Additionally, there was no significant difference between groups in peak twitch force (*p* = 0.22), time to peak twitch force (*p* = 0.24), or half-relaxation time (*p* = 0.52) at baseline (Table 2). However, younger women had significantly larger M-wave amplitude (*p* = 0.001) at baseline than older women (Table 2). Older women had significantly longer cortical silent period duration (*p* = 0.02) and significantly larger MEP amplitude (*p* = 0.05) at baseline (*p* = 0.02) than young women (Table 2).

### 3.2. Mental Fatigue

**Figure 3:**
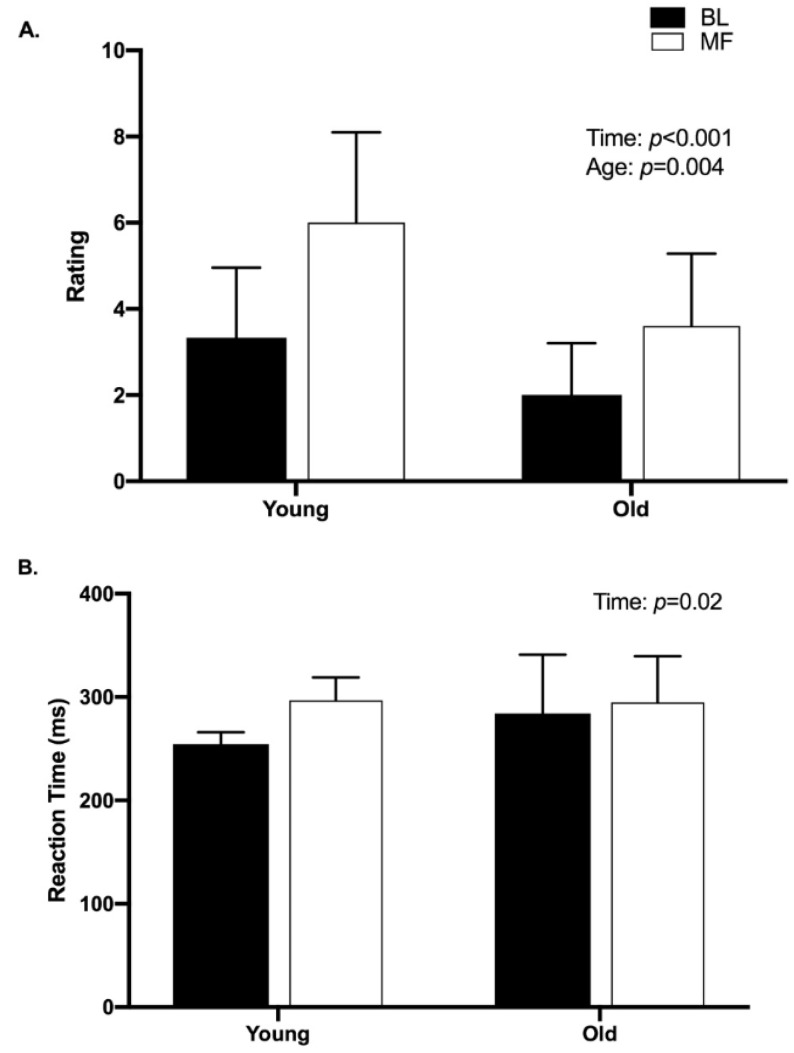
(**A**) Subjective fatigue ratings. Higher values indicate higher feelings of fatigue. After the mental fatigue task (MF), ratings were significantly higher (*p* < 0.001) than ratings at baseline (BL). Older women reported lower fatigue than younger adults (*p* = 0.004). (**B**) Psychomotor vigilance task reaction time. MF times were significantly slower than BL (*p* = 0.02).

Likert ratings of subjective fatigue are shown in Figure 3A. There was a significant main effect of time (*p* < 0.001; *d* = 5.0), as ratings after mental fatigue were significantly higher than ratings at baseline. Additionally, there was a significant main effect of age (*p* = 0.004, *d* = 2.2), with older women reporting lower fatigue than younger women overall. However, there was no significant interaction of time and age (*p* = 0.30). 

There was a significant main effect of time for reaction time (pre versus post mental fatigue) (*p* = 0.02, *d* = 7.6), with the post-mental fatigue time point having significantly longer reaction times than baseline (Figure 3B). However, there was no significant main effect of age (*p* = 0.47, *d* = 0.53) for reaction time on the PVT, nor was there a significant interaction of time and age (*p* = 0.15).

False starts and lapses are presented in Table 3. There was a significant difference between groups (*p* = 0.01) on number of false starts, with older women having significantly more false starts than young women. There were significantly more false starts at the beginning of the task, compared with the end (*p* = 0.02), however there was no significant group × time interaction (*p* = 0.45). There was no significant difference between groups (*p* = 0.13), or across time (*p* = 0.66) in the number of lapses, nor was there a significant group × time interaction (*p* = 0.08).

### 3.3. Force

There was a significant main effect of time on force (*p* = 0.02, *d* = 1.9), with lower MVC force values post mental fatigue, compared with baseline (Figure 3A). However, there was no significant main effect of age (*p* = 0.32, *d* = 0.5) on MVC force, nor was there a significant interaction of time and age (*p* = 0.09). The force, relative to MVC, at the end of the mental fatigue protocol was not significantly different (*p* = 0.19, *d* = 0.08) between young and older women (Figure 3B).

### 3.4. Neuromuscular Measurements

There was no significant main effect of age or time, respectively, on peak twitch force (*p* = 0.29, *p* = 0.10), time to peak twitch force (*p* = 0.73, *p* = 0.24), or half-relaxation time (*p* = 0.49, *p* = 0.22) (Table 2). There was also no significant interaction of time and age for peak twitch force (*p* = 0.62), time to peak (*p* = 0.07), or half-relaxation time (*p* = 0.81). 

Young women had significantly larger amplitude M-waves (*p* = 0.002) than older women (Table 2). However, there was no significant main effect of time (*p* = 0.09), nor was there a significant interaction of time and age (*p* = 0.83).

Older women had significantly longer CSP durations (*p* = 0.03) than young women (Table 2). There was a trend toward an increase in CSP duration over time, but this failed to reach statistical significance (*p* = 0.06). There was no significant interaction of time and age (*p* = 0.48). 

There was a significant main effect of age on MEP amplitude (*p* = 0.04) with older women having larger MEP amplitudes than young (Table 2). There was no significant main effect of time (*p* = 0.69) on MEP amplitude, nor was there a significant interaction of time and age (*p* = 0.14).

## 4. Discussion

The purpose of this study was to examine potential age-related differences in the effect of mental fatigue on neuromuscular function. To our knowledge, this is the first study to include assessments of both central and peripheral neuromuscular function in response to mental fatigue. Furthermore, the comparison of such responses in young and older adults is novel and important to our understanding of age-related functional changes. The results of this study suggest that mental fatigue may influence the ability to produce force in young adults. After the mental fatigue task, young females produced significantly less force than at baseline. Contrary to our hypothesis, neuromuscular function measures did not change with mental fatigue. However, there was a trend toward increased cortical inhibition after mental fatigue.

### 4.1. Baseline Measurements

As sleep quality can affect the PVT [28], the PSQI was administered to determine if there were any differences in sleep quality between groups. There was no significant difference between age groups sleep ratings, indicating that at baseline, there was no difference between groups in sleep quality. However, older women reported significantly higher amounts of subjective fatigue on the Multidimensional Fatigue Index. This agrees with results found by Schwarz et al. [29], who also found that older adults scored significantly higher on the MFI than young adults. 

The TA muscle plays an important functional role in age-related differences in dynamic balance control [30]. We therefore chose to study this muscle, as impacts of mental fatigue on the control of this muscle may have important functional implications, particularly in older adults. There was no significant difference between young and older women in ability to produce force. Several studies have reported similar results in the ankle dorsiflexors [31,32,33]. For example, McNeil et al. [33,34] found no difference in MVC torque in the TA between young (23–32 years) and older (61–69 years) men. However, the same group of studies had a “very old” group (80–90 years) of men who were significantly weaker than the young men. The older women in the present study were fairly young (~74 years) and since no differences in strength were found, this may suggest that major strength changes in the TA do not occur until much later in life [32,33]. The lack of difference between age groups may be explained by the muscle fiber composition of the tibialis anterior; the TA is composed of 70%–80% Type I muscle fibers [35,36,37] and Type I fibers seem to be less affected by aging [37]. 

There were no significant differences in contractile properties of the muscle (TTP, PTF, HRT) between age groups, suggesting that contractile properties in the TA may be unaffected by aging. However, older women had significantly smaller amplitude M-waves. These results are in agreement with a study by Klass et al. [38] who demonstrated no difference in peak twitch torque or half-relaxation time but significantly smaller amplitude M-waves in the tibialis anterior in older adults compared with young. Changes in the neuromuscular system with aging such as decreases in muscle fiber number and size, as well as decreases in conduction velocity with aging could contribute to a decreased M-wave amplitude in older adults, while not producing substantial changes in the contractile properties [39,40]. 

Older women had significantly longer duration CSP (~27 ms) than younger women. These results are similar to those in a study examining age related changes in intracortical properties in the flexor carpi radialis of young and older adults in which researchers found that older adults had significantly longer duration CSP (~28 ms) than younger adults [41]. In the current study, older women had significantly larger MEPs than young adults, suggesting higher cortical excitability at baseline. This is similar to results found by Bernard and Seidler [42], who showed larger amplitude MEPs in the first dorsal interosseous muscle in older adults compared with young. Higher levels of cortical excitability in the older adults could be a compensation for previously discussed declines in the aging neuromuscular system as well as increased cortical inhibition. Previous research exploring motor cortical representation and brain region recruitment in older adults indicates that older adults have more dispersed motor cortical representation and less specific patterns of brain region recruitment when performing cognitive tasks, which could lead to a larger MEP amplitude in older adults [42,43].

### 4.2. Mental Fatigue and Force Output

Twenty minutes of the PVT induced a significant increase in both subjective fatigue rating and reaction time, indicating the presence of mental fatigue. This is consistent with previous studies that used the PVT to induce mental fatigue [26,28]. There was no difference between age groups in reaction time, but older women reported lower levels of subjective fatigue overall. This may suggest that while both groups experienced mental fatigue (overall increase in reaction time and subjective fatigue rating) younger women felt more fatigued (higher subjective fatigue ratings in young women). 

Force output in the mentally fatigued state was significantly lower than in the baseline condition and this was not different between age groups. This suggests that mental fatigue affects physical function but not differently between young and older women. It is interesting to note that older women experienced only a 7% decrease in MVC force with mental fatigue while young women experienced a 16% decrease in MVC force. We did not observe significant changes with mental fatigue in contractile properties of the muscle (TTP, PTF, HRT), or in M_Max_. Therefore, we can assume that changes in force output in both groups with mental fatigue are more likely caused by supraspinal mechanisms (suboptimal output from the motor cortex [44]) than by mechanisms at the muscle or in neuromuscular transmission. 

Changes in cortical function may help to explain the significant decrease in force observed in both groups. The increase in CSP duration after mental fatigue (young 8.7%, older 13%) approached significance (*p* = 0.055), indicating that an increase in cortical inhibition may have been present. Dopamine has been shown to lengthen the duration of the CSP [45,46] and a study using positron emission tomography has demonstrated an attention related increase in dopamine release during a sustained attention task [47]. Therefore, the increase in CSP duration in the present study could be attributed to an increased release of dopamine during the PVT, which is a sustained attention task. However, further work is necessary to determine whether dopamine is responsible for the observed changes in CSP duration, and if such a mechanism is influenced by advanced age.

Older women tended to have increased MEP amplitudes after mental fatigue (7%) whereas young women tended to have decreased MEP amplitudes (−17%). Though not statistically significant, the tendency for reduced excitability in the young women may help to explain the reduced MVC force observed in that group following mental fatigue. In contrast, older women may have compensated for the increased inhibition from mental fatigue through an increase in cortical excitability (though not significant) to maintain corticomotor drive to the muscle. 

Although the exact mechanism of mental fatigue leading to a decline in MVC force remains unclear at this point, changes in adenosine concentrations offer another possible explanation. There is evidence to suggest that adenosine, released during the performance of a cognitive task, can increase perceived exertion and reduce motivation leading to decreased physical performance [48]. In animal studies, adenosine resulted in both reduced performance on the rat PVT [49] and running endurance [50]. Furthermore, caffeine (adenosine antagonist) decreases perceived exertion in humans during whole body exercise [51]. While the exact mechanism is not known, it has been demonstrated that prolonged neural activity causes release of adenosine and subsequent suppression of excitatory transmission [52]. Martin and colleagues [48] proposed that local accumulation of adenosine, an inhibitory neurotransmitter, creates a need for greater stimulatory signals from motivational brain centers, such as the anterior cingulate cortex (ACC), to produce motor output. The ACC is involved in performance monitoring, effort/reward, and perceived exertion [48] and performance on the PVT is associated with activity in this region of the brain. In the present study, therefore, prolonged performance of the PVT may have led to increased local levels of adenosine in the ACC leading to both increased subjective ratings of fatigue and decreased MVC force output. However, it is important to note that no measure of adenosine was recorded in this study and this concept warrants further research.

### 4.3. Limitations

It has been suggested that older women report greater levels of fatigue than men [53]. For this reason we chose to focus this study on women. The results therefore may not be generalizable to men, and future work, with larger sample sizes, should further explore potential sex differences in the impact of mental fatigue. Furthermore, as noted above, older women reported greater levels of fatigue than young on the MFI. As state levels of general fatigue are associated with mental fatigue [20], it is possible that these baseline differences influenced the results, although the effect size was low (*d* = 0.21). However, given this association, we would expect older adults to have a larger change in Likert ratings of fatigue, and reaction time and number of lapses on the PVT, which was not the case. It is therefore unlikely that the general state of fatigue, as assessed by the MFI, impacted the results of the current study. Finally, TMS-based measures of excitability (MEP) in particular can be impacted by several factors including contraction intensity [54] and hormone levels [55] and contractions intensity. We chose a contraction intensity of 50% MVC to help reduce variability in the MEP amplitude [54]. However, we did not control for the phase of the mensural cycle in young women, which may have impacted the MEP results. Further work is necessary to fully understand the impact of hormonal influences, and the interactions with age, on the effects of mental fatigue. Measures of spinal excitability would also be beneficial for the further interpretation of MEP results. 

## 5. Conclusions

Results from the present study suggest that 20 min of a mentally fatiguing task may cause a decrease in the ability to produce maximal force in both young and older women. Significant decreases in MVC force were seen in both groups, and the decrease was not different between young and older women. Increases in the cortical silent period duration approached significance in both young and older groups, suggesting that mental fatigue may cause increased cortical inhibition. Measures of peripheral neuromuscular function (contractile properties of the muscle, M_Max_) did not change, suggesting that changes in force production with mental fatigue are more likely due to supraspinal than peripheral mechanisms. To our knowledge, this is the first study using TMS to examine the effects of mental fatigue on neuromuscular function. These findings provide further evidence of an interaction between mental fatigue and physical function [4,6,7], and provide novel information suggesting that the underlying neuromuscular mechanisms are similar between young and older women.

## Figures and Tables

**Table 1:** Participant characteristics.

	YW (*n* = 9)	OW (*n* = 16)	ES (*d*)
Age, year *	22.4 ± 2.9	74.1 ± 6.3	8.5
Height, in	65.4 ± 3.4	64.1 ± 2.1	0.29
Weight, kg	56.2 ± 12.8	60.5 ± 8.8	0.25
MFI *	48.1 ± 25.5	55.0 ± 14.9	0.21
PSQI	5.3 ± 2.8	4.5 ± 2.1	0.21
SFR	3.3 ± 1.6	2.0 ± 1.2	0.59

Values are means ± SD. *n*, no. of participants; YW, young women; OW, older women; MFI, Multidimensional Fatigue Index; PSQI, Pittsburgh Sleep Quality Index; SFR, subjective fatigue rating; CSP, cortical silent period; MEP, motor evoked potential; M_Max_, maximal M-wave; ES, effect size. Higher values indicate more fatigue or poorer sleep quality. * Significant difference between age groups *p* < 0.05.

**Table 2:** Neuromuscular measures before and after mental fatigue.

	YF (*n* = 9)		OF (*n* = 16)			
	Baseline	Mental Fatigue	Baseline	Mental Fatigue	ES (*d*) Age	ES (*d*) Time
PTF, N	13.7 ± 5.5	12.0 ± 2.6	16.5 ± 2.2	13.5 ± 5.0	0.56	0.61
TTP, ms	86.4 ± 16.8	88.8 ± 13.5	94.9± 7.7	84.8 ± 12.0	0.18	0.31
HRT, ms	98.5 ± 10.9	89.0 ± 14.8	105.9 ± 23.0	91.8 ± 23.7	0.28	0.65
M_Max_, mv *	6.0 ± 1.2	5.6 ± 1.3	4.0 ± 0.8	3.5 ± 1.2	1.8	0.40
CSP, ms	74.4 ± 6.3	81.4 ± 9.7	101.4 ± 27.0	114.7 ± 30.2	1.6	0.56
MEP, %M_Max_	27.8 ± 8.0	23.0 ± 7.9	42.9 ± 15.2	45.8 ± 17.1	1.6	0.08

Values are means ± SD. PTF, peak twitch force; TTP, time to peak twitch force; HRT, half relaxation time; M_Max_, m-wave; MEP, motor evoked potential; CSP, cortical silent period; ES, effect size. * indicates a significant difference between ages at baseline *p* = 0.001.

**Table 3:** False starts and lapses during the PVT.

	YF (*n* = 9)		OF (*n* = 16)			
	Baseline	Mental Fatigue	Baseline	Mental Fatigue	ES (*d*) Time	ES (*d*) Age
False Starts * †	0.38 ± 0.52	0.25 ± 0.46	2.13 ± 1.5	0.75 ± 1.18	1.2	0.76
Lapses	0.13 ± 0.35	0.63 ± 1.06	1.44 ± 1.5	0.63 ± 1.36	0.61	0.16

Values are means ± SD. PVT, psychomotor vigilance task; *n*, no. of participants; YW, young women; OW, older women ES, effect size. * Significant difference between age groups *p* = 0.01; **†** Significant difference across time *p* = 0.02.
